# Thoracic spinal manipulation: exploring force-time profiles and force modulation strategies in French chiropractors

**DOI:** 10.1186/s12998-026-00649-9

**Published:** 2026-05-12

**Authors:** Mégane Pasquier, Nathalie Thurnherr, Wanda Dupèbe, Lindsay M. Gorrell, Martin Descarreaux, Arnaud Lardon

**Affiliations:** 1 Institut Franco-Européen de Chiropraxie, Ivry-sur-Seine, France; 2 Department of Chiropractic Medicine, Integrative Spinal Research Group, Balgrist University Hospital, University of Zurich, Zurich, Switzerland; 3 Department of Human Kinetics, Université du Québec à Trois-Rivières, Trois- Rivières, Québec Canada

**Keywords:** Motor control, Force modulation, Biomechanical parameters, Manual therapy, Expertise

## Abstract

**Background:**

Spinal manipulation (SM) involves the application of controlled, high-velocity low-amplitude (HVLA) forces with specific biomechanical properties. While prior research has outlined how these force-time characteristics evolve with practitioner expertise, the strategies used to intentionally adjust such parameters remain insufficiently explored. This study sought to assess the thoracic SM profiles of French chiropractors and to explore how instructions related to a clinical scenario, age, gender and years of practice are associated with SM force-time profiles.

**Methods:**

Forty-six licensed chiropractors participated during a national professional gathering on the 15th and 16th March, 2024. Each performed thoracic HVLA manipulations on a manikin under three sequential conditions: initial familiarization, a simulated clinical case involving a patient with thoracic pain, and a constrained task requiring a 50% reduction in peak force. A force-sensing table captured detailed force-time data, including preload, peak force, peak thrust duration, and rate of force application. Linear mixed models were performed to examine the effects of trial number and experimental condition on each force-time parameter, with subjects included as random effects to account for repeated measures.

**Results:**

In the clinical simulation, the average peak force reached 569.1 N, with an average rate of force application of 3093.8 N/s. During the modulation task, chiropractors applied significantly lower forces (mean peak: 379.0 N) and a reduced rate of force application (mean: 2072.8 N/s). Clinical scenarios and gender significantly impacted SM biomechanical parameters (*p* < 0.05). No effect was observed for age, year of practice, or types of therapy techniques.

**Conclusion:**

Chiropractors adapted their force during the 50% modulation task by substantially reducing peak force and rate of force application, while minimally decreasing peak thrust duration This pattern could reflect a pulse-height control strategy, where force amplitude is scaled while temporal structure is preserved, suggesting that training consistent timing may enhance precision and adaptability in manual therapy.

## Introduction

Motor expertise can be defined as the specialized knowledge and perceptual motor skills acquired through deliberate practice over an extended period of time [1]. It is characterized by the ability to perform given tasks with precision and efficiency, leading to improved decision-making, refined motor skills, and superior information-processing capabilities [2]. Therefore, individuals who achieve motor expertise, whether they are athletes, specialized workers or health professionals, demonstrate a higher level of motor awareness within their specific domains. For instance, in a study investigating various objective measures of robotic surgical performance, Judkins et al. (2009) found that expert surgeons were significantly better than novices in several objective measures such as task completion time, speed, and amplitude of gesture [3].

Manual therapy skills such as spinal manipulation (SM) have been described in the literature using various theoretical definitions and biomechanical features [4]. SM can be described as a treatment delivered to the spinal segment structures and involving a force applied with specific parameters of angulation, amplitude, and speed to an intervertebral articulation, resulting in distinct biomechanical and neurophysiological effects [5]. SM is characterized by its unique force-time profile involving different force events, including: peak force, peak thrust duration, preload force, rate of force application, and total force delivered [6].

Differences between SM characteristics of novices and experts have also been explored in several studies by comparing students to clinicians [7–9]. These studies have highlighted specific differences in how students and experienced clinicians deliver SM following comparisons of peak force, thrust duration, rate of force application, and general force-time profiles. Overall, when comparing novices to experienced clinicians, the latter show SM force-time profile characterized by a lower peak thrust duration [8, 9], a higher rate of force application, less variability during execution and better reproduction of the SM procedure [8, 10]. Previous studies indicate that basic components of SM (peak thrust duration, peak force and rate of force application) [9, 10] are mastered sequentially by students in such a way that, by the end of their training, they achieve performance levels for these basic components that are similar to experienced clinicians. More advanced components, however, such as general coordination [8], error detection [11], transfer capabilities [7] and retention [8, 10] seem to develop later, during the first years of clinical practice [12].

Expertise development is believed to be a long-term process involving hours of deliberate practice and resulting in maximum adaptation to specific task-constraints [1]. In a narrative review of the effect of age and expertise on fine motor movement, Kamper (2002) reported that professional musicians demonstrated superior maximum finger-tapping rates compared to musically untrained or amateur musicians [13]. Additionally, trained musicians exhibited better timing accuracy and reduced variability in movement implementation compared to novices. Although force modulation capacities can reflect expertise, how chiropractors successfully achieve such modulation through changes in basics SM parameters remains unknown.

Therefore, the objectives of the present study were [1] to quantify thoracic SM force-time profiles in a sample of French chiropractors and [2] and to explore how instructions related to a clinical scenario, age, gender and years of practice are associated with SM force-time profiles.

## Methods

### Study design

This cross-sectional study was conducted in accordance with the Strengthening the Reporting of Observational Studies in Epidemiology (STROBE) guidelines. The ethics committee of the Institut Franco-Européen de Chiropraxie (IFEC) approved this study with certificate CER-24_01_01.

### Participants

A total of 46 chiropractors registered to a French regional health agency, on the 25th March 2024, voluntary participated in this study. Exclusion criteria were pain or injury that did not allow the participant to perform SM. Written informed consent was obtained from each participant.

### Equipment

All SM trials were performed on a Human Analogue Mannequin (HAM^®^) (Canadian Memorial Chiropractic College (CMCC), Toronto, Ontario, Canada) used in previous SM learning studies [10]. All force-time parameters of the SM were recorded by a Force Sensing Table Technology^®^ system (FSTT^®^, CMCC, Toronto, ON), composed of a Leander 900 Z Series Chiropractic Table (Leader Health Technologies Corporation, Lawrence, KS) and an embedded force plate (AMTI, Watertown, MA). All transmitted forces were computed in a XYZ coordinate system using the FSTT^®^ software (CMCC, Toronto, ON).

### Experimental procedures

The study was set during an annual convention of the French chiropractic association in March 2024. The project was briefly presented by a member of the research team in a plenary lecture. Volunteers were invited to go to the High Velocity Low Amplitude (HVLA) laboratory where a member of the research team provided all information about the research project. After reading the informed consent form, participants had the possibility to ask questions. Researchers checked inclusion/exclusion criteria and asked participants to complete a Google form questionnaire regarding their demographics (gender (women/men), age (in years), years of practice (number of years), type of therapy techniques [manual therapy techniques used in the participants’ daily clinical practice (e.g., manual-only techniques, instrument-assisted techniques)], chiropractic school [institution from which participants earned their chiropractic degree], weight and height).

For all participants, the experiment was organized in three different blocks. Three familiarization trials followed by two blocks of five SM trials on a manikin were conducted (Block 1: familiarization; Block 2: clinical simulation; and Block 3: a 50% decrease peak force modulation). During block 1 (familiarization), three SM trials were performed on a manikin by participants using a HVLA postero-to-anterior thoracic SM technique of their choice. Participants were asked to use the same technique throughout the different blocks. Visual feedback with all the SM biomechanical parameters and oral feedback with peak of force in Newtons were provided to the participant.

Then, during block 2 (clinical simulation), participants were asked to deliver five SM at the force that they usually implement in daily practice in the following situation: “A 31-year-old man, pillar in the local rugby team, 1.76m and 96 kg, presents with non-specific middle back pain. After clinical examination, you discover/found/palpate thoracic hypomobility. There are no contraindications to the delivery of SM. You decide to perform a SM at the T8 level with the postero-to-anterior HVLA technique of your choice.”

Finally, during block 3 (50% modulation), participants were asked to reduce peak force by 50% for five trials based on the force applied during the previous block. To do so, the following instructions were provided: “For the next series of five thrusts, please perform the same technique as before, but reduce the force you apply. Your goal is to deliver each thrust with about 50% less peak force compared to the thrusts you performed in the previous block.

Focus only on reducing the overall force you apply during the thrust; do not try to intentionally change any other aspect of your technique”. There was no feedback provided during blocks 2 and 3. Participants performed five trials in each block (2 and 3). The inter-trial time interval within each block was approximately 60 s, allowing sufficient time for repositioning and equipment resetting. The inter-block time interval between blocks 2 and 3 was approximately 3 min, providing participants with a short rest period. Participants were not instructed to report any trial they judged as an error or not representative of their intended performance.

### Data acquisition

**Fig. 1:**
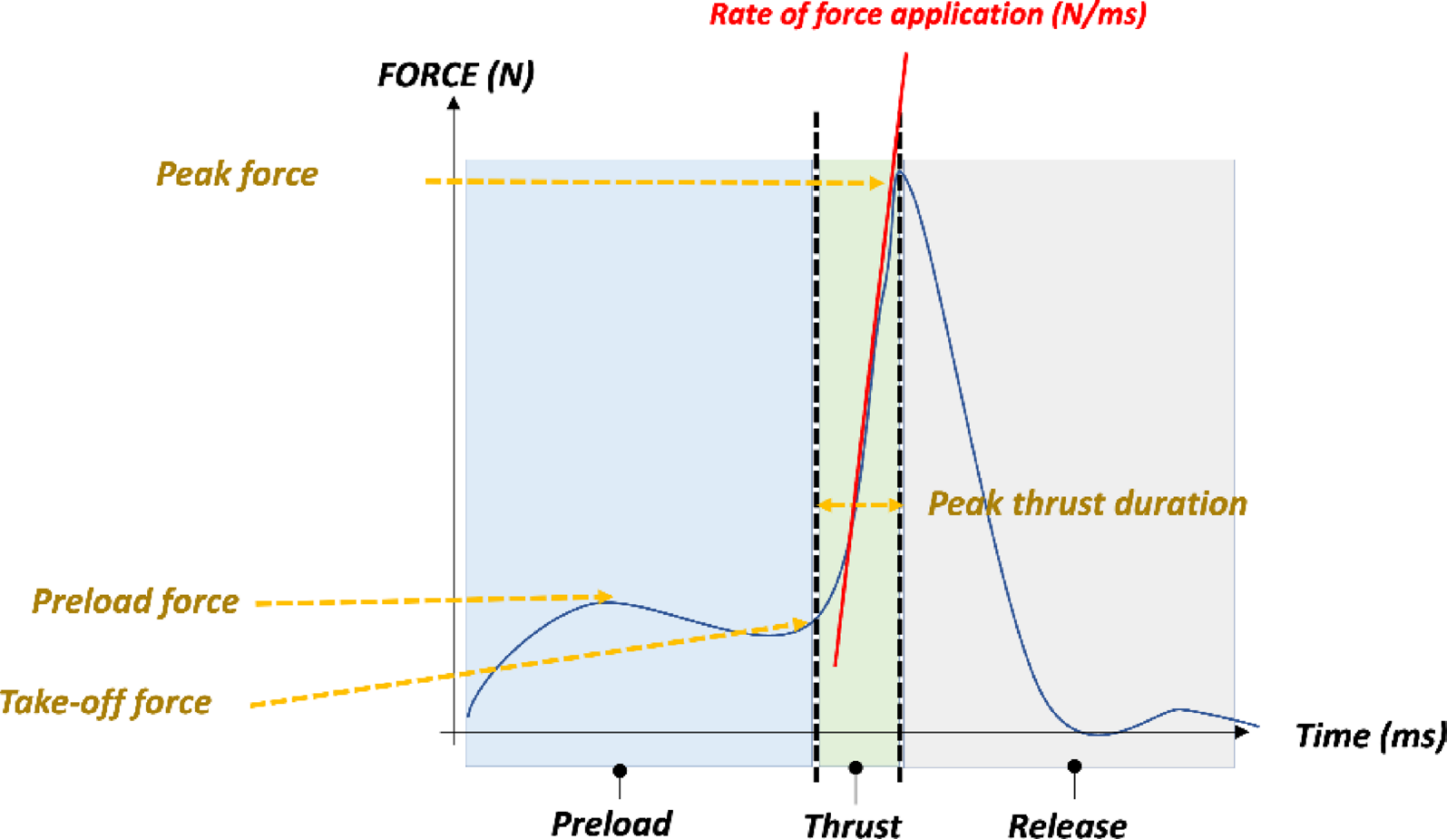
Force time profile and phases of a spinal manipulation and biomechanical parameters

The vertical force-time signals (Fz) applied to the manikin during HVLA manipulation were recorded for each trial. SM biomechanical parameters (i) Preload force (N): the average force applied prior to the thrust, ii) Peak force (N): the maximum force value of the thrust, including any force applied during the preload phase, iii) Peak thrust duration (ms): the time elapsed from takeoff force to peak force, iv) Rate of force application (N/ms): the average change in applied force between the take-off force and peak force were analyzed. Details are presented in Fig. 1.

### Sample size

A sample size calculation was conducted for the planned Linear Mixed Model analysis using peak force as the primary outcome. Based on prior data, peak force was assumed to have a mean of 450 N with a standard deviation of 150 N [4]. Assuming a Type I error rate of 0.01 and 90% statistical power, the required total sample size was estimated at 37 participants. The calculation was based on the expected effect size and the assumed variance–covariance structure of the repeated measures.

### Statistical methods

#### Descriptive statistics

Mean participants’ demographics (age, gender, years of practice, height and weight) and force-time parameters of SM for block 2 and 3 (preload force, peak force, peak thrust duration, rate of force) were calculated.

#### Linear mixed model

All statistical analyses including sample size calculation were performed using R (R Core Team, 2023). Outliers for each variable of interest were identified using the interquartile range method (rstatix package) and extreme values (beyond 3×IQR) were removed. To identify the most relevant covariates to include in the statistical models, we first compared several candidate mixed-effects models using the Akaike Information Criterion (AIC) and Bayesian Information Criterion (BIC). These indices allowed us to evaluate model fit while penalizing unnecessary complexity, and thus guided the selection of the most parsimonious set of fixed effects. After determining the optimal model structure, we fitted a separate linear mixed-effects model for each biomechanical outcome using the lmer function (lme4 package), with participant identification number specified as a random intercept to account for repeated measurements within individuals. The fixed effects included gender (women/men), age (in years), years of practice (number of years), type of therapy techniques [manual therapy techniques used in the participants’ daily clinical practice (e.g., manual-only techniques, instrument-assisted techniques)], chiropractic school [institution from which participants earned their chiropractic degree], trial number, and conditions (Block 2 vs. block 3).

The statistical results are presented using the following terms: for a fixed effect, estimate (β) indicates the estimated coefficient for each fixed effect, SE refers to the standard error of the estimate t-value is the test statistic for the fixed effect, p-value (or t-based threshold for REML models) indicates statistical significance and finally 95% CI represents the 95% confidence interval of the estimate.

## Results

### Participant demographics

A total of 46 chiropractors (23 women and 23 men) participated in the study. The participants’ mean age was 39.5 (± 11.9) years, with a mean practice experience of 14.8 (± 11.2) years. The majority of chiropractors (89.13%) obtained their diploma from IFEC, while others graduated from Université du Québec à Trois-Rivières (UQTR) (4.35%), CMCC (2.17%), National University of Health Sciences (2.17%) and Western States Chiropractic College (2.17%). HVLA manipulation was reported to be the main technique used in clinical practice (86.96%), whereas instrumentally and mechanically assisted manipulation were less commonly reported (21.94% and 19.56% respectively). Detailed information regarding participant demographics is presented in Table 1.

**Table 1:** Participants demographics

Gender (*n* women/*n* men)	23/23
Age, years (mean (*± SD))*	39.5(± 11.9)
Years of practice, (years/ mean (*± SD))*	14.8(± 11.2)
Weight, kg (mean (*± SD))*	72.8 (± 14.0)
Height, m (mean (*± SD))*	1.72 (± 0.09)
Chiropractic School Institut Franco-Européen de Chiropraxie, n (%) Université du Québec à Trois-Rivières, n (%) Canadian Memorial Chiropractic College, n (%) National University of Health Sciences, n (%) Western States Chiropractic College, n (%)	41 (89.13%) 2 (4.35%) 1 (2.17%) 1 (2.17%) 1 (2.17%)
HVLA manipulation utilisation in clinical practice 76–100%, n (%) 51–75%, n (%) 26–50%, n (%) 1–25%, n (%) Never, n (%)	31 (67.39%) 9 (19.57%) 3 (6.52%) 2 (4.35%) 1 (2.17%)

### SM biomechanical characteristics

In the simulated clinical scenario (block 2), as presented in Table 2, chiropractors applied a mean peak force of 569.1 N (± 142.1) on the manikin. The preload force used was 169.8 N (± 83.1) and the thrust duration was 132.3ms (± 22.8). The mean rate of force application was 3093.8 N/ms (± 909.9) during SM.

During the 50% modulation condition (block 3), chiropractors exerted a mean peak force of 379.0 N (± 95.6) on the manikin. The associated preload force was 140.3 N (± 58.4) with a peak thrust duration of 118.3ms (± 25.4). The mean rate of force application was 2072.8 N/ms (± 615.7) during the SM. Due to technical issues, data for two participants were not computed, which explains why 44 participants were analyzed for block 3.

**Table 2:** SM biomechanical parameters

	Block 2	Block 3
	Clinical simulation (*n* = 46)	50% modulation (*n* = 44)
Preload force *(N)*; mean(± SD*)*, range	169.8 (± 83.1), 5.2-321.8	140.3 (± 58.4), 21.4–278.0
Peak force *(N)*; mean(± SD*)*, range	569.1 (± 142.1), 230.2-1003.4	379.0 (± 95.6), 193.8–631.0
Peak thrust duration *(ms)*; mean(± SD*)*, range	132.3 (± 22.8), 98.8-213.8	118.3 (± 25.4), 79.4–218.0
Rate of force application *(N/ms)*; mean(± SD*)*, range	3093.8 (± 909.9), 1313.4-6495.6	2072.8 (± 615.7), 893.4-3681.8

N, Newtons; ms, millisecond; SD, standard deviation

### Linear mixed model effects

#### Peak force

**Fig. 2:**
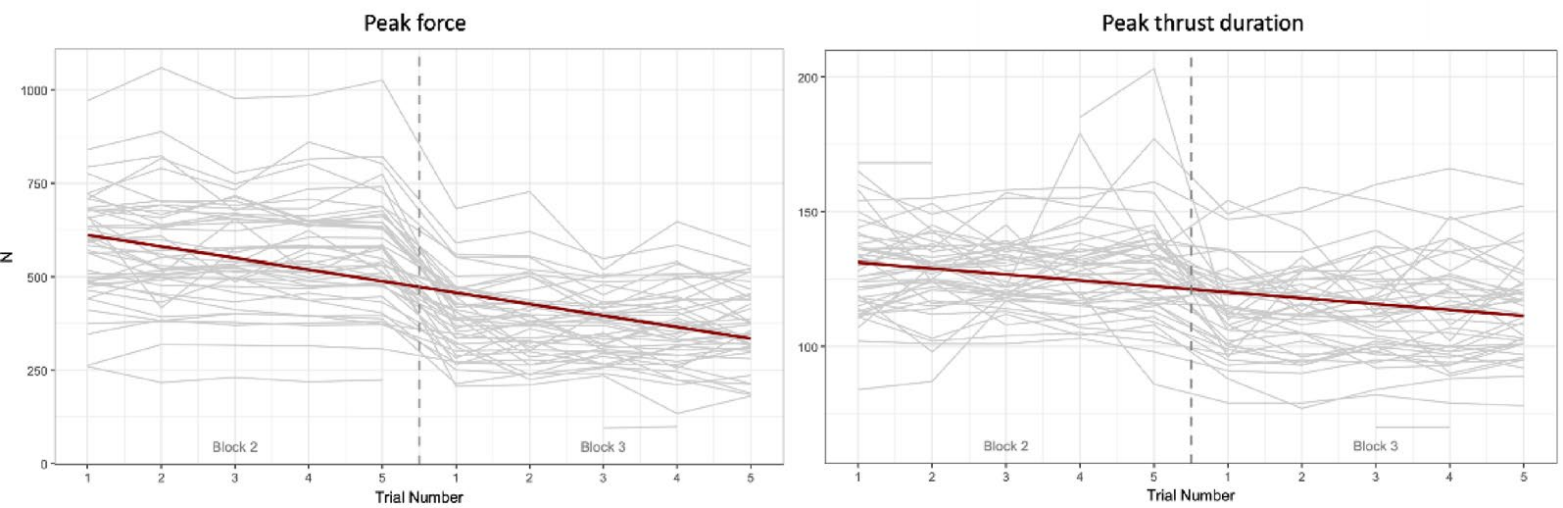
Linear regression of peak force and peak thrust duration across trial and blocks. Peak force (left) is in Newtons (N) and peak thrust duration in millisecond (ms)

**Fig. 3:**
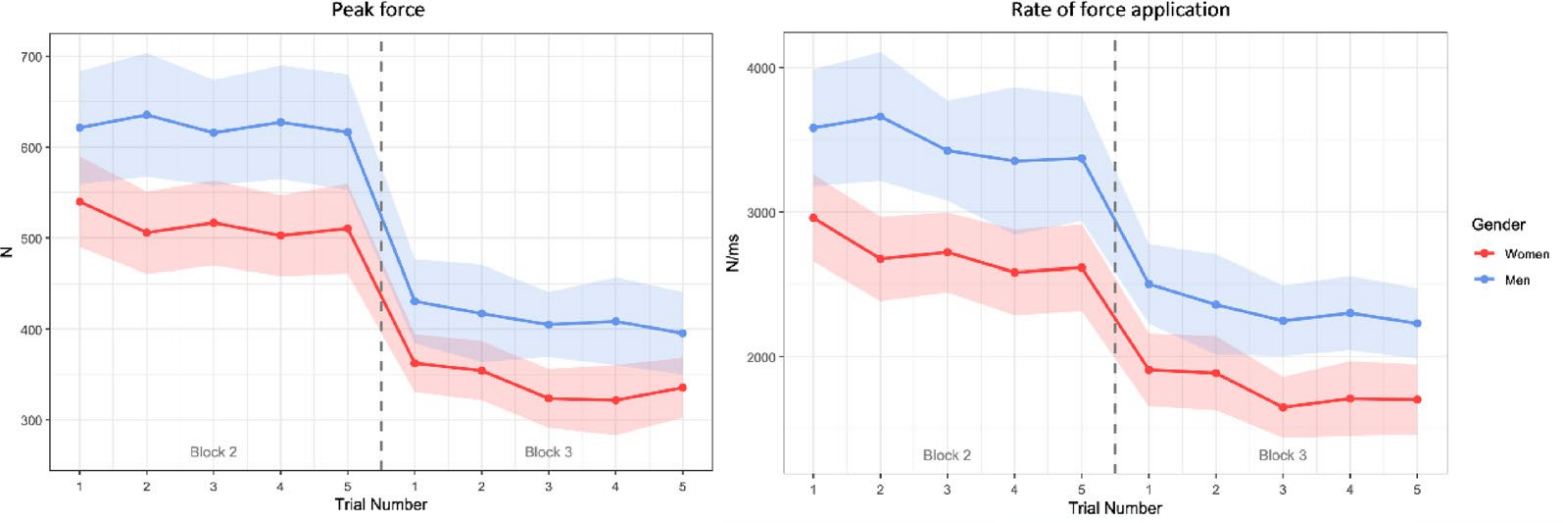
Linear regression plots depicting the relationship between trial number and peak force (left) and rate of force application (right), separated by gender. Lines represent fitted regression models, and shaded bands correspond to 95% confidence intervals. The dashed vertical line denotes the transition from block 2 to block 3

The linear mixed model revealed a significant main effect of the clinical scenarios on peak force, with participants producing substantially lower forces during block 3 compared with block 2 (β = −197.83, SE = 5.30, t = − 37.36, *p* < 0.001) (Fig. 2). A significant effect of gender was also observed (β = 103.80, SE = 33.56, t = 3.09, *p* = 0.004) (Fig. 3), with males producing higher peak force. A small but significant effect of trial was observed (β = −5.86, SE = 1.87, t = − 3.14, *p* = 0.002). No other fixed effects were statistically significant (all *p* > 0.05).

#### Peak thrust duration

Peak thrust duration significantly decreased between block 2 and 3 (β = −15.07, SE = 0.97, t = − 15.55, *p* < 0.001). No significant effects were found for gender, age, diploma, trial number, or type of therapy technique (all *p* > 0.05).

Rate of Force Application.

A significant effect of clinical scenario was observed (β = −1074.0, SE = 44.6, t = − 24.08, *p* < 0.001) as well as a significant effect for gender (β = 645.0, SE = 216.2, t = 2.98, *p* = 0.006) (Fig. 3), trial number (β = −67.82, SE = 15.72, t = − 4.31, *p* < 0.001) and therapy techniques used (β = −661.2, SE = 280.6, t = − 2.36, *p* = 0.025). All other fixed effects were nonsignificant (*p* > 0.05).

#### Preload force

Preload force was significantly reduced between blocks (β = −48.27, SE = 3.38, t = − 14.28, *p* < 0.001). No other fixed effects reached statistical significance, and all remaining predictors were nonsignificant (*p* > 0.05).

## Discussion

The overall aims of this study were to quantify thoracic SM force-time profiles of French chiropractors and to explore how clinical scenarios, age, gender and years of practice are associated with SM force-time profiles. Our sample consisted of women and men that mostly graduated from IFEC. The majority of participants identified HVLA SM as their main technique with limited use of instrumentally and mechanically assisted manipulation. Results of the study showed that clinical scenarios and gender significantly impacted SM biomechanical parameters. No effect was observed for age, year of practice, or types of manipulation techniques.

### Force time profiles

One objective of this study was to quantify the ability of French chiropractors to modulate thoracic force-time profiles using a single clinical scenario. When treating a 31-year-old male rugby player (clinical simulation), French chiropractors delivered force-time profiles similar to another study who used a picture of a 30-year-old healthy male athlete for the clinical simulation [14]. In this previous study, 81 chiropractors performed thoracic SM on a manikin and the average peak force was 694.95 N (± 248.24), with a preload force average of 173.00 N (± 105.43) [14]. However, such forces are greater than those reported in the literature in which SM was delivered to live humans, without the use of imagined clinical scenarios. In fact, a scoping review reporting on force-time characteristics of SM, including SM delivered to the thoracic spine, described peak force values ranging from 337 N to 536 N, with a preload force between 137 N and 172 N and an peak thrust duration ranging from 87ms to 198ms when measuring forces at the manikin-table interface [4]. The higher peak forces observed in our study compared with those reported in live human subjects likely reflect the use of a manikin and a simulated scenario. Without the constraints of patient feedback, pain, or tissue compliance, chiropractors may apply greater forces, highlighting the influence of context on force modulation. This interpretation is supported by recent work showing that experienced orthopaedic manual therapy physiotherapists apply systematically higher forces when delivering thoracic mobilization and manipulation to a manikin compared with human recipients, while still modulating force according to different clinical scenarios in both conditions. As a matter of fact, Thurnherr at al. (2025) explore whether the force–time characteristics of mobilization and manipulation performed by experienced physiotherapists to the thoracic spine of a manikin were different from those delivered to healthy humans. The authors recruited three patients that could match each clinical scenario used on the manikin where scenario 1 was a young male (30 years, 185 cm), scenario 2 a middle-aged mal (50 years, 175 cm) and scenario 3 an elderly female (65 years, 165 cm). Peak forces applied during spinal manipulation were higher in the manikin rather in human, for example, respectively it was observed for scenario 1: peaks forces at 483 N vs. 355 N, scenario 2: 490 N vs. 444 N or scenario 3: 421 N vs. 284 N. Their findings suggest that clinicians are able to adapt force output to contextual cues, but that the mechanical properties and tactile fidelity of the manikin substantially influence the magnitude of applied forces [15]. Additionally, it could suggest that inter-practitioner variability may arise from differences in experience, technique, or motor strategies.

### Force modulation

Nyirö’s et al. (2024) study showed the importance of clinical scenarios. In their study, three different clinical scenarios were provided to chiropractors before the delivery of SM to: (i) a 50-year-old healthy male patient, (ii) a 70-year-old healthy female patient, and (iii) a 30-year-old healthy male athlete [14]. In these three scenarios, patients all presented with uncomplicated thoracic pain. As expected, the highest peak force was reported for the 30-year-old athlete (694.95 *N* ± 248.24) and the lowest force for the 70-year-old female (406.32 *N* ± 187.88), indicating that practitioners modulated the applied SM force according to the patient’s profile. Triano et al. (2015) investigated the stability of SM performance on manikin in terms of peak force development over time and clinicians’ ability to adapt to different force targets (normal/typical, half-force, and double-force conditions). When asked to perform half their “typical” force, participants applied 14% more than the target (i.e., they overshot the target) and could not reduce their force sufficiently [16] as observed in the present study.

Similarly to Nyirö’s et al. (202*4)* results, our study failed to identify associations between age, years of practice and differences in peak force modulation capacity. However, in contrast to their findings, gender emerged as a significant predictor of several SM biomechanical parameters in our study, specifically peak force, rate of force application, with men producing higher forces than women. Importantly, no gender difference was observed in thrust duration, indicating that gender-related differences influenced force amplitude but not the temporal structure of the thrust. Despite these baseline differences, both men and women similarly reduced their peak force between blocks, with only minimal changes in thrust duration or velocity. This pattern suggests that practitioners predominantly modulated force amplitude rather than timing parameters. These findings extend observations by Pasquier et al. (2019), who reported substantial inter-practitioner variability in thoracic SM force–time characteristics among French chiropractors. While their study did not examine gender effects, the variability they observed is consistent with our results showing that individual characteristics (gender-related differences in force production) can partly explain differences in SM performance [17]. However, like Pasquier et al. (2019), we also found that experience-related variables did not meaningfully predict biomechanical outcomes.

Finally, Descarreaux et al. (2015) investigated the association between expertise and transfer capabilities. Their results suggest that SM biomechanical parameters are modulated by expertise as well as performance conditions such as preferred position, table height, and unstable surfaces. Specifically, the results showed a decreased thrust duration and increased rate of force application in experienced clinicians, whereas thrust force and rate of force application were significantly decreased when task difficulty was increased. Increasing task difficulty also led to significant increases in performance variability [7]. However, our study failed to identify an association between years of practice and SM biomechanical parameters, suggesting that under simple, stable task conditions, individual motor capabilities (including gender-related differences in strength or rapid force generation) may play a more prominent role than accumulated clinical experience.

SM parameter modulation was characterized by a marked reduction in applied force, preload force, and rate of force application during the clinical scenario describing the 50% force modulation task. This modulation profile was defined by a substantial decrease in peak force and rate of force application, while peak thrust duration was only slightly reduced (approximately 9%, 132.3 ms vs 118.3 ms. This result is consistent with the concept of force modulation capacity described by Gordon and Ghez (1987), whereby the central nervous system can scale the amplitude of force output without substantially altering its temporal structure, a strategy known as ‘’pulse height control’’ [18]. More recent evidence suggest that ballistic forces often exceed intended forces. Miyamoto et al. who observed a discrepancy between intended and produced ballistic forces concluded that ballistic contractions are generated by brief, preprogrammed neural commands whose amplitude (pulse height) determines the resulting force [19]. Overshooting in the context of ballistic force application, such as during SM force production, is therefore not unexpected. Forces were then potentially modulated in the following trials, as the central nervous system adjusted the pulse height, gradually reducing overshoot and refining the force output towards the initial goal, despite limited sensory guidance.

### Limitations

The sample size of 46 participants, although providing a substantial number of manipulations per participant, is unlikely to be fully representative of the broader chiropractic practitioner population in France (about 3% of all registered chiropractors). While the linear mixed-effects model robustly accounts for within-participant variability and trial-to-trial effects, caution is still needed when generalizing the findings to the broader population. The use of a manikin rather than human subjects introduces a specific limitation, as it does not fully reflect the tactile properties of the human body. Therefore, the mechanical responses of a manikin may differ from those of human patients, potentially affecting the applicability of the results to clinical settings. Further research is needed to establish a more comprehensive understanding of SM force applications and modulation mechanisms and impacts, particularly in complex and changing clinical environments involving human patients.

### Practical application

This study could help to develop learning strategies and environments that foster predictable and consistent force application patterns. The linear mixed-effects analysis revealed trial-to-trial variability and learning/fatigue effects, highlighting the importance of repeated practice with feedback to stabilize force-time profiles for novice practitioners. In the learning process of SM, keeping rise time consistent allows novice practitioners to develop more predictable and controlled force-time profiles, which is essential for reproducibility and accuracy. Drawing on the principles of force rise time regulation observed by Gordon and Ghez, practitioners could be trained to accelerate SM skill learning and potentially improve clinical practice. For instance, and as part of a global motor learning strategy, task-specific training using relevant feedback can be employed to reinforce the rise time modulation capacity. Although clear evidence linking SM force parameters to safety is lacking, practicing consistent force application allows students and even clinicians to refine coordination, reduce variability, and potentially deliver more effective and safer therapy.

## Conclusion

In this study, the force application patterns and overall SM biomechanical parameters observed in French chiropractors were consistent with those previously reported in the literature under comparable experimental clinical scenario. When asked to reduce SM force, participants were able to modulate forces by primarily reducing the peak force and rate of force application, while maintaining the peak thrust duration. Additionally, the results indicate that instructions related to the clinical scenario and gender significantly influenced several SM biomechanical parameters. In contrast, no significant effects were observed for age, years of practice, or type of manipulation technique. Strengthening clinicians’ ability to modulate SM forces especially by preserving consistent timing while scaling amplitude may enhance precision, adaptability to clinical demands, and the refinement of SM motor skills.

## Data Availability

The datasets generated and analyzed during the current study are available from the corresponding author on reasonable request.

## References

[1] Ericsson KA. Deliberate practice and acquisition of expert performance: a general overview. Acad Emerg Med Off J Soc Acad Emerg Med nov. 2008;15(11):988–94. 10.1111/j.1553-2712.2008. 00227.x PubMed PMID: 18778378. 10.1111/j.1553-2712.2008.00227.x 18778378

[2] Dukas R. Cognitive innovations and the evolutionary biology of expertise. Philos Trans R Soc B Biol Sci 23 oct. 2017;372(1735):20160427. 10.1098/rstb.2016.0427. 10.1098/rstb.2016.0427 PMC5665814 29061899

[3] Judkins TN, Oleynikov D, Stergiou N. Objective evaluation of expert and novice performance during robotic surgical training tasks. Surg Endosc mars. 2009;23(3):590–7. 10.1007/s00464-008-9933-9 . PubMed PMID: 18443870. 10.1007/s00464-008-9933-9 18443870

[4] Gorrell LM, Nyirö L, Pasquier M, Pagé I, Heneghan NR, Schweinhardt P, et al. Spinal manipulation characteristics: a scoping literature review of force-time characteristics. Chiropr Man Ther 13 sept. 2023;31(1):36. 10.1186/s12998-023-00512-1 . PubMed PMID: 37705030; PubMed Central PMCID: PMC10500795. 10.1186/s12998-023-00512-1 PMC10500795 37705030

[5] Pagé I, Descarreaux M. Effects of spinal manipulative therapy biomechanical parameters on clinical and biomechanical outcomes of participants with chronic thoracic pain: a randomized controlled experimental trial. BMC Musculoskelet Disord déc. 2019;20(1):29. 10.1186/s12891-019-2408-4. 10.1186/s12891-019-2408-4 PMC6339327 30658622

[6] Herzog W. The biomechanics of spinal manipulation. J Bodyw Mov Ther juill. 2010;14(3):280–6. 10.1016/j.jbmt.2010. 03.004 PubMed PMID: 20538226. 10.1016/j.jbmt.2010.03.004 20538226

[7] Descarreaux M, Dugas C, Treboz J, Cheron C, Nougarou F. Learning Spinal Manipulation: The Effect of Expertise on Transfer Capability. J Manipulative Physiol Ther mai. 2015;38(4):269–74. 10.1016/j.jmpt.2015.02.001. 10.1016/j.jmpt.2015.02.001 25925020

[8] Marchand AA, Mendoza L, Dugas C, Descarreaux M, Pagé I. Effects of practice variability on spinal manipulation learning. J Chiropr Educ oct. 2017;31(2):90–5. 10. 7899/JCE-16-8 PubMed PMID: 28121458; PubMed Central PMCID: PMC5656152. 10.7899/JCE-16-8 PMC5656152 28121458

[9] Colloca CJ, Cunliffe C, Hegazy MA, Pinnock M, Hinrichs RN. Measurement and Analysis of Biomechanical Outcomes of Chiropractic Adjustment Performance in Chiropractic Education and Practice. J Manipulative Physiol Ther. 2020;43(3):212–24. 10.1016/j.jmpt.2019.05.006 . PubMed PMID: 32709512. 32709512 10.1016/j.jmpt.2019.05.006

[10] Pasquier M, Memari S, Lardon A, Descarreaux M. Can self-assessment and augmented feedback improve performance and learning retention in manual therapy: results from an experimental study. Chiropr Man Ther 12 sept. 2023;31(1):35. 10.1186/s12998-023-00505-0. 10.1186/s12998-023-00505-0 PMC10498620 37700344

[11] Loranger M, Treboz J, Boucher JA, Nougarou F, Dugas C, Descarreaux M. Correlation of expertise with error detection skills of force application during spinal manipulation learning*. J Chiropr Educ 1 mars. 2016;30(1):1–6. 10.7899/JCE-15-4. 10.7899/JCE-15-4 PMC4770989 26270897

[12] Descarreaux M, Dugas C. Learning spinal manipulation skills: assessment of biomechanical parameters in a 5-year longitudinal study. J Manipulative Physiol Ther. 2010;33(3):226–30. .011 PubMed PMID: 20350677. 20350677 10.1016/j.jmpt.2010.01.011

[13] Krampe RT. Aging, expertise and fine motor movement. Neurosci Biobehav Rev nov. 2002;26(7):769–76. 10.1016/S0149-7634(02)00064-7. 10.1016/s0149-7634(02)00064-7 12470688

[14] Nyirö L, Gorrell LM, Cecchini V, Menon C, Elgendi M, Schweinhardt P. Variability and repeatability of spinal manipulation force-time characteristics in thoracic spinal manipulation on a manikin. Chiropr Man Ther 11 nov. 2024;32(1):33. 10.1186/s12998-024-00551-2 . PubMed PMID: 39529165; PubMed Central PMCID: PMC11552221. 10.1186/s12998-024-00551-2 PMC11552221 39529165

[15] Thurnherr N, Schweinhardt P, Gorrell LM. Force variability of thoracic spine mobilization and manipulation delivered by experienced physiotherapists to healthy human volunteers and a manikin: an observational study. Chiropr Man Ther 9 déc. 2025;33(1):56. 10.1186/s12998-025-00619-7 . PubMed PMID: 41366446; PubMed Central PMCID: PMC12690789. 10.1186/s12998-025-00619-7 PMC12690789 41366446

[16] Nougarou F, Dugas C, Deslauriers C, Pagé I, Descarreaux M. Physiological responses to spinal manipulation therapy: investigation of the relationship between electromyographic responses and peak force. J Manipulative Physiol Ther. 2013;36(9):557–63. .006 PubMed PMID: 24161387. 24161387 10.1016/j.jmpt.2013.08.006

[17] Pasquier M, Barbier-Cazorla F, Audo Y, Descarreaux M, Lardon A. Learning spinal manipulation: Gender and expertise differences in biomechanical parameters, accuracy, and variability*. J Chiropr Educ 1 mars. 2019;33(1):1–7. 10.7899/JCE-18-7. 10.7899/JCE-18-7 PMC6417869 30408423

[18] Gordon J, Ghez C. Trajectory control in targeted force impulses. II. Pulse height control. Exp Brain Res. 1987;67(2):241–52. 10. 1007/BF00248546 PubMed PMID: 3622687. 3622687 10.1007/BF00248546

[19] Miyamoto T, Kizuka T, Ono S. The Influence of Contraction Types on the Relationship Between the Intended Force and the Actual Force. J Mot Behav. 2020;52(6):687–93. 10.1080/00222895.2019.1680947 . PubMed PMID: 31665979. 31665979 10.1080/00222895.2019.1680947

